# Increased Levels of NF-kB-Dependent Markers in Cancer-Associated Deep Venous Thrombosis

**DOI:** 10.1371/journal.pone.0132496

**Published:** 2015-07-20

**Authors:** Grazia Malaponte, Salvatore S. Signorelli, Valentina Bevelacqua, Jerry Polesel, Martina Taborelli, Claudio Guarneri, Concettina Fenga, Kazou Umezawa, Massimo Libra

**Affiliations:** 1 Department of Biomedical and Biotechnological Sciences, Section of General & Clinical Pathology and Oncology, University of Catania, Catania, Italy; 2 Department of Clinical and Experimental Medicine, University of Catania, Medical Angiology Unit, Garibaldi Hospital, Catania, Italy; 3 Epidemiology and Biostatistics Unit, Centro di Riferimento Oncologico-National Cancer Institute, Aviano, Italy; 4 Department of Clinical and Experimental Medicine, Section of Dermatology, University of Messina, Messina, Italy; 5 Department of Environmental and Health Sciences (S.A.S.T.A.S.), University of Messina, Messina, Italy; 6 Department of Molecular Target Medicine Screening, Aichi Medical University, Nagakute, Japan; INRS, CANADA

## Abstract

Several studies highlight the role of inflammatory markers in thrombosis as well as in cancer. However, their combined role in cancer-associated deep vein thrombosis (DVT) and the molecular mechanisms, involved in its pathophysiology, needs further investigations. In the present study, C-reactive protein, interleukin-6 (IL-6), tumor necrosis factor-α (TNF-α), interleukin-1 (IL-1β), matrix metalloproteases-9 (MMP-9), vascular endothelial growth factor (VEGF), tissue factor (TF), fibrinogen and soluble P-selectin, were analyzed in plasma and in monocyte samples from 385 cancer patients, of whom 64 were concomitantly affected by DVT (+). All these markers were higher in cancer patients DVT+ than in those DVT-. Accordingly, significantly higher NF-kB activity was observed in cancer patients DVT+ than DVT-. Significant correlation between data obtained in plasma and monocyte samples was observed. NF-kB inhibition was associated with decreased levels of all molecules in both cancer DVT+ and DVT-. To further demonstrate the involvement of NF-kB activation by the above mentioned molecules, we treated monocyte derived from healthy donors with a pool of sera from cancer patients with and without DVT. These set of experiments further suggest the significant role played by some molecules, regulated by NF-kB, and detected in cancer patients with DVT. Our data support the notion that NF-kB may be considered as a therapeutic target for cancer patients, especially those complicated by DVT. Treatment with NF-kB inhibitors may represent a possible strategy to prevent or reduce the risk of DVT in cancer patients.

## Introduction

The relative risk of developing deep venous thrombosis (DVT) is approximately seven times higher in patients with cancer [1,2] suggesting a bidirectional correlation between thrombosis and inflammation in cancer. Chemotherapy is one of the most important risk factors for increased risk of DVT [3,4]. Thrombosis and cancer are linked by numerous pathophysiological mechanisms that are generally related to the host response to cancer. These mechanisms include activation of the coagulation and fibrinolytic systems, acute phase reaction, inflammation, and cytokine production [5]. The systemic activation of coagulation that occurs in malignancy is well known and has been described under the name of Trousseau’s syndrome [6,7]. Systemic inflammation is a potent prothrombotic stimulus leading to an up-regulation of procoagulant factors, down regulation of anticoagulants and inhibition of fibrinolytic activity [8,9]. Chronic inflammation is often associated with increased risk of cancer [10,11]. Rudolf Virchow demonstrated the presence of leukocytes in tumors and suggested that tumors arise at sites of chronic inflammation and that inflammatory mediators, by enhancing cell proliferation, may serve as tumor promoters [12]. Inflammatory cells, cytokines in malignant tumors affect the stromal microenvironment, suggesting that inflammation and cancer may be interrelated through the angiogenic process [13–15]. During inflammation, angiogenesis often coincides with the infiltration of inflammatory cells such as neutrophils, monocytes/ macrophages, which secrete cytokines and growth factors [13,16,17]. It was shown that numerous mediators play a critical role in inflammation, cancer and thrombosis such as C-reactive protein, interleukin-6 (IL-6) and tumor necrosis factor-α (TNF-α), interleukin-1β (IL-1β) (markers of inflammation), matrix metalloproteases-9 (MMP-9), vascular endothelial growth factor (VEGF) (reflecting angiogenesis), tissue factor (TF) and fibrinogen (coagulation markers) and soluble P-selectin (marking platelet activation). Inflammatory cytokines up-regulate various angiogenic factors, such as VEGF and MMP-9, in vascular endothelial cells, cancer cells, and monocytes/macrophages [18–35]. Monocytes participate in the pathological processes of inflammation and thrombosis through their ability to synthesize TF and expressing P-selectin upon stimulation [36–38]. Tissue factor (TF), which is the primary cellular initiator of blood coagulation, contributes to the tumor-related pathological processes, such as hypercoagulability, tumor growth, angiogenesis, and metastasis [39–40]. Intriguingly, all of these molecules are regulated by NF-kB [41,42]. This is an inducible transcription factor controlled by the signal activation cascades. NF-kB controls a number of genes involved in inflammatory responses, cell cycle progression, inhibition of apoptosis and cell adhesion, thus promoting tumor angiogenesis, carcinogenesis and cancer progression. Prevention and management of DVT in cancer patients can significantly affect patient treatment, prognosis, and quality of life. Therefore, there is a need to identify novel bio-molecular markers that can recognize cancer patients with high risk of DVT. Although several studies investigated on the role of different bio-markers and/or cytokines in cancer and/or thrombosis, no previous studies have analyzed altogether these NF-kB-regulated markers that in turn regulate NF-kB itself in cancer patients with and without thrombosis. The identification of these markers may recognize NF-kB as an appealing target for therapeutic intervention.

In the present study a fraction of NF-kB-regulated markers have been measured in peripheral blood from cancer patients with and without DVT. Moreover, the effects of dehydroxymethylepoxyquinomicin (DHMEQ), a NF-kB inhibitor [43,44], were evaluated to demonstrate the direct role of the NF-kB-regulated markers in thrombosis development among cancer patients. Furthermore, the identification of biomolecular markers of DVT in cancer patients may support the significance of pharmacological thromboprophylaxis.

## Methods

### Study population

Peripheral blood samples from three groups of subjects were collected in the last 10 years at the Department of Bio-medical Sciences, University of Catania, Catania, Italy. These groups included: 64 cancer patients with concomitant DVT (DVT+) (mean age 64 ±10 years); 321 cancer patients with no history of DVT (DVT-) (mean age 62 ± 9 years); 100 healthy controls (mean age 61± 12 years), matched by sex and age (Table 1). Patients were diagnosed as affected by the DVT based on the no compression of a deep vein of lower limbs by doppler probe and/or the by the presence of the echogenic pattern into a deep veins of the lower limbs. DVT patients were recruited at the time of the DVT occurrence. Peripheral blood samples from these patients were obtained before the assumption of any specific antithrombotic drug. Detailed information on the study were given to the patients. All patients gave a written informed consent prior to enrolment. Our study was approved by the University of Catania ethics committee. All procedures were conducted in accordance with the principles outlined in the Declaration of Helsinki. All the individuals in this manuscript have given written informed consent (as outlined in PLOS consent form) to participate in this study. Patients treated with anti-inflammatory drugs, statins and anticancer compounds such as, bevacizubam, thalidomide, lenalidomide and/or radiation therapy were excluded. Other exclusion criteria included: BMI > 35 kg/m^2^, severe systemic depression, diabetes, dyslipidemias, hypertension, chronic inflammatory diseases, heart failure at different stage, mild or severe renal failure (creatinine ≥ 2.0 mg/dl), pancytopenia and more recent surgical intervention (≤ 3 months). Venous blood samples were collected at least after 3 months of the last anticancer treatment for both cancer patients DVT+ and DVT-.

**Table 1:** Characteristics of controls and cancer patients with and without deep vein thrombosis. Abbreviations: DVT, deep vein thrombosis; BMI, body mass index.

	Controls (n = 100) %		Cancer (n = 321) %		Cancer DVT+ (n = 64) %		
**Age** (Years, mean ± SD)	60±10		62±9		64±10		p = 0.07^$^
**Sex**							
Male	57	(57)	150	(46.7)	35	(54.7)	p = 0.14^^^
Female	43	(43)	171	(53.3)	29	(45.3)	
**Smoking habits**							
Yes	53	(53)	156	(48.6)	37	(57.8)	p = 0.35^^^
No	47	(47)	165	(51.4)	27	(42.2)	
**BMI** (kg/m^2^, mean ± SD)	24.5±6.8		25.3±7.3		26.4±8.3		p = 0.25^$^
**Metastasis**							
No			181	(56.4)	29	(45.3)	p = 0.10^^^
Yes			140	(43.6)	35	(54.7)	
**Cancer type or site**							
Lung			47	(14.6)	7	(10.9)	p = 0.14^^^
Breast			53	(16.5)	6	(9.4)	
Gastrointestinal			101	(31.5)	31	(48.4)	
Genitourinary			41	(12.8)	6	(9.4)	
Hematologic			34	(10.6)	8	(12.5)	
Other			45	(14)	6	(9.4)	

^$^Evaluated through analysis of variance (ANOVA).

^^^Evaluated through χ^2^ test.

### Blood collection and laboratory procedures

Blood was collected from each patient and healthy controls drawn in to pyrogen-free blood collection tubes with and without additives. Citrated platelet-poor plasma was made using two centrifuge steps: 5 min at 4000 r.p.m and 10 min at 11000 r.p.m. Multiple aliquots of serum and plasma were stored at—80°C. Blood samples were utilized for the following analyses: 1) CRP, Fibrinogen, IL-6, TNF-α, IL-1β, MMP-9, VEGF, TF antigen and sP-selectin plasma levels; 2) monocytes isolation. Plasma fibrinogen levels and high-sensitivity C-reactive protein were measured with standard techniques used in the Central Laboratory of Catania University Hospital. IL-6, TNF-α, IL-1β, MMP-9, VEGF and sP-selectin were measured by enzyme-linked immunosorbent assays (ELISAs; R&D Systems Europe, Abingdon, Oxfordshire, UK). Plasma TF antigen level was measured by ELISA using a commercially available kit, IMUBIND (American Diagnostica Inc, Stamford, CT). All assay procedures were performed according to the manufacturer’s protocol. Control specimens were analyzed simultaneously on each plate for every marker.

### Reagents

Ficoll Histopaque 1077, RPMI 1640 medium, penicillin-streptomicin solution, L-glutamin, fetal bovin serum (FBS), phosphate-buffered saline solution (PBS), Percoll, lipopolysaccharide (LPS) from *Escherichia coli* serotype 0128:B12, MTT (3-[4,5-Dimethylthiazol-2-yl]-2,5-diphenyltetrazolium bromide; Thiazolyl blue) and Dimethylsulphoxide (DMSO) were purchased from Sigma Chemical (St. Louis, MO, USA). Iscove's medium and Polymixin B from Gibco, Life Technologies Inc (Milan, Italy).

DHMEQ (dehydroxymethylepoxyquinomicin), synthesized as previously described, [43] was dissolved in dimethylsulfoxide (DMSO) at a concentration of 10 μg/mL and stored at 20°C. This stock solution was diluted in culture medium to a final concentration of <0.1%. Agents. DHMEQ was kindly provided by Dr Kazuo Umezawa, Department of Applied Chemistry, Faculty of Science and Technology, Keio University, Yokohama, Japan.

### Isolation of human monocytes

Peripheral blood mononuclear cells (PBMC) were isolated from citrated blood of cancer patients DVT+ and DVT- and healthy controls by Ficoll-Paque. For isolation of monocytes, PBMC were placed for 2 h in culture plate, and the non-adherent cells were removed with three changes of warm PBS. Pure monocytes were then positively selected by anti-CD14-coated magnetic microbeads (Mini MACS separation column; Milteny Biotec, Bergisch Gladbach, Germany) following manufacturer’s instructions. Monocyte purity was > 98%, as assessed by flow cytometry (data not shown). The cells were pooled and resuspended in a final concentration of 1x10^6^ cells/ml in a freeze medium consisting of 30% autologous citrated plasma, 60% Iscove's and 10% DMSO. Next the cells were frozen in aliquots of 1 ml in sterile cryovials (Greiner, Germany) using a standard controlled freezing procedure and then stored in liquid nitrogen.

### Treatment and culturing of human monocytes

After thawing at room temperature, the cryopreserved monocytes had a viability of 85%, as shown by trypan blue exclusion. Human monocytes were analyzed for NF-kB activation, for cytokines production and for TF activity assay (see succeeding text). Briefly, monocytes (5 x10^5^/mL) were harvested, washed, and seeded onto wells in RPMI 1640 supplemented with 10% heat-inactivated FBS, L-glutamine (2 mM), penicillin (100 IU/mL), streptomycin (100 μg/mL), and pretreated with DHMEQ (10 μg/mL) for 2 hours and after which incubated for 24 hours. Next, cell culture supernatants were collected for quantification of cytokine protein level by an enzyme-linked immunosorbent assay (ELISA). Endotoxin contamination of cell cultures was routinely excluded with the chromogenic limulus amebocyte lysate assay (Sigma). Furthermore, in all cell cultures 10 μg/ml of polymixin B was added to neutralize any potential LPS contamination.

### ELISA for active NF-kB p65 subunit

For measurement NF-kB p65 subunit activation, nuclear extracts were prepared from 5x10^5^ monocytes, pretreated and not with DHMEQ (10 μg/mL) for 2 h and after which stimulated with 100 ng/ml LPS for the desired period of time, using a Nuclear Extract Kit (Active Motif, Rixensart, Belgium) according to the manufacturer’s protocol. Levels of nuclear p65 concentrations were determined by a sensitive ELISA assay (TRANS-AM, Active Motif, Rixensart, Belgium).

### Detection of TF activity in monocytes by a chromogenic assay

In cell lysates the activity of TF was measured by actichrome1 TF activity kit (American diagnostic, Pfungstadt, Germany) according to manufacturer’s instructions. In brief. samples were coincubated with factor VII and spectrozyme fVIIa. TF-FVIIa complex cleaves spectrozyme fVIIa as a highly specific chromogenic substrate releasing a paranitroanilin-chromophore with a specific change of absorption at 405 nm. Results are expressed in picomoles per litre (pM) of peptidyl activity of lipidated TF cleaving the spectrozyme fVIIa complex.

### Serum-dependent activation of cytokines and NF-kB in monocytes

Monocytes (5x10^5^) from 25 healthy donors were cultured for 20 hours in medium (RPMI 1640) supplemented with either 40% serum from 64 cancer patients DVT+ and 257 DVT- with the highest cytokines plasma levels (≥ 75^th^ percentile) or 40% serum from 100 healthy donors with the lowest cytokines values (≤ 25^th^ percentile). For processing of serum, 80μl of serum from each cancer patient DVT+ and DVT- and from each healthy donor was added to the monocyte culture medium immediately after thawing. The cells were treated with DHMEQ (10 μg/mL) for 2 h, and after which stimulated with 100 ng/mL LPS for the desired period of time. Treatment of monocytes with LPS was used as positive control. In the LPS experiments no polymixin B was added. After incubation, cell culture supernatants were also collected for quantification of cytokine protein level and NF-kB activation by ELISA.

### MTT assay

Effects of DHMEQ on cell viability were assayed by the 3-(4.5-dimethylthiazol-2-yl)-2.5-diphenyl tetrasodium bromide (MTT) method [45]. The monocytes (3x10^5^) were seeded in 96-well plates for 48 hours for cytostatic DHMEQ. Serial dilutions of DHMEQ (at 2 μg/mL, 5 μg/mL, 10 μg/mL) were generated and added to the cells. After incubation with DHMEQ or DMSO at the indicated concentrations and time points. Cells treated by MTT solution (1mg/mL) for 4 hours were measured by a microplate reader (Bio-Rad, Richmond, CA) at a reference wavelength of 630 nm and a test wavelength of 570 nm. The cell viability was expressed as a percentage of the DMSO-treated control samples.

### Statistical analysis

For continuous variables, difference in mean between study groups was evaluated through the analysis of variance (ANOVA, in the case of three groups) or t-test (two groups). For discrete variables, the difference in distribution across study groups was evaluated through the test χ^2^ test. The correlation between continuous variables was evaluated through the Spearman rank correlation coefficient *r*.

## Results

Baseline characteristics of healthy controls and cancer patients with and without DVT are shown in Table 1. No significant differences were observed among groups with respect to age, sex, current smoking, and body mass index. The frequency of localized cancers was similar among cancer patients with and without DVT, as well as the distribution of cancer type/site (Table 1).

### Biomarker plasma levels of inflammation, angiogenesis and coagulation in cancer patients DVT+ and DVT-

Mean plasmatic levels of different biomarkers, including those of inflammation, angiogenesis and coagulation, analyzed in the control group and in cancer patients with and without DVT are reported in Table 2. Compared to control group, higher plasma levels of CRP, fibrinogen, IL-6, TNF-α, IL-1β, MMP-9, VEGF, TF antigen, and sP-selectin were observed in cancer patients with and without DVT (P<0.01). As expected, the concentration of all markers in DVT+ group was significantly higher than in DVT- (p<0.01) with the only exception of fibrinogen (p = 0.35). Correlation of all markers with each other are reported in (S1 Table). Positive correlations (|r|>0.45) among CRP, IL-6, TNF-α, IL-1β, MMP-9, VEGF, and TF antigen were found in both groups of cancer patients, whereas the fibrinogen correlated only in DVT+ group. No correlation among markers were observed in healthy controls (S1 Table).

**Table 2:** Plasma levels of inflammation, angiogenic and coagulation markers from controls and cancer with and without deep vein thrombosis. Abbreviations: DVT, deep vein thrombosis; IL-6, interleukin-6; TNF- α, tumor necrosis factor-α; IL-1β, interleukin-1β; CRP, C-reactive protein; MMP-9, matrix metalloproteinase-9; VEGF, vascular endothelial growth factor; TF, tissue factor; soluble P-selectin (sP-selectin). Significant differences among inflammatory, angiogenic and coagulation markers were evident in cancer patients with and without DVT compared to controls with further increments in DVT cancer patients. P value are given using analysis of variance (ANOVA test, in the case of three groups) or t-test (two groups).

	Controls (n = 100)	Cancers (n = 321)	Cancers DVT+ (n = 64)	Controls *vs* Cancers *vs* Cancer DVT+	Cancers *vs* Cancer+DVT
	Mean ± SD	Mean ± SD	Mean ± SD		
**Inflammation markers**					
IL-6	3.7 ± 1.5	11.5 ± 3.9	16.7 ± 4.9	p<0.01	p<0.01
TNF-α	2.6 ±1.3	8.5 ± 3.7	10.6 ± 3.4	p<0.01	p<0.01
IL-1β	1.5 ± 0.7	5.5 ± 1.8	9.4 ± 2.5	p<0.01	p<0.01
CRP	0.1 ± 0.1	0.8 ± 0.7	1.6 ± 1.1	p<0.01	p<0.01
**Angiogenesis markers**					
MMP-9	43.0 ± 16.1	153.1 ± 50.5	204.3 ± 60.4	p<0.01	p<0.01
VEGF	60.1 ± 33.2	322.7 ± 125.1	439.3 ± 126.8	p<0.01	p<0.01
**Procoagulant markers**					
Fibrinogen	246.4 ± 47.2	404.2 ± 71.1	413.7 ± 87.7	p<0.01	p = 0.35
TF	30.4 ± 7.8	141.1 ± 34.0	184.5 ± 41.4	p<0.01	p<0.01
sP-selectin	36.4 ± 11.5	55.4 ± 22.5	75.8 ± 32.3	p<0.01	p<0.01

### Cytokines and MMP-9 secretion in human monocytes

**Fig 1:**
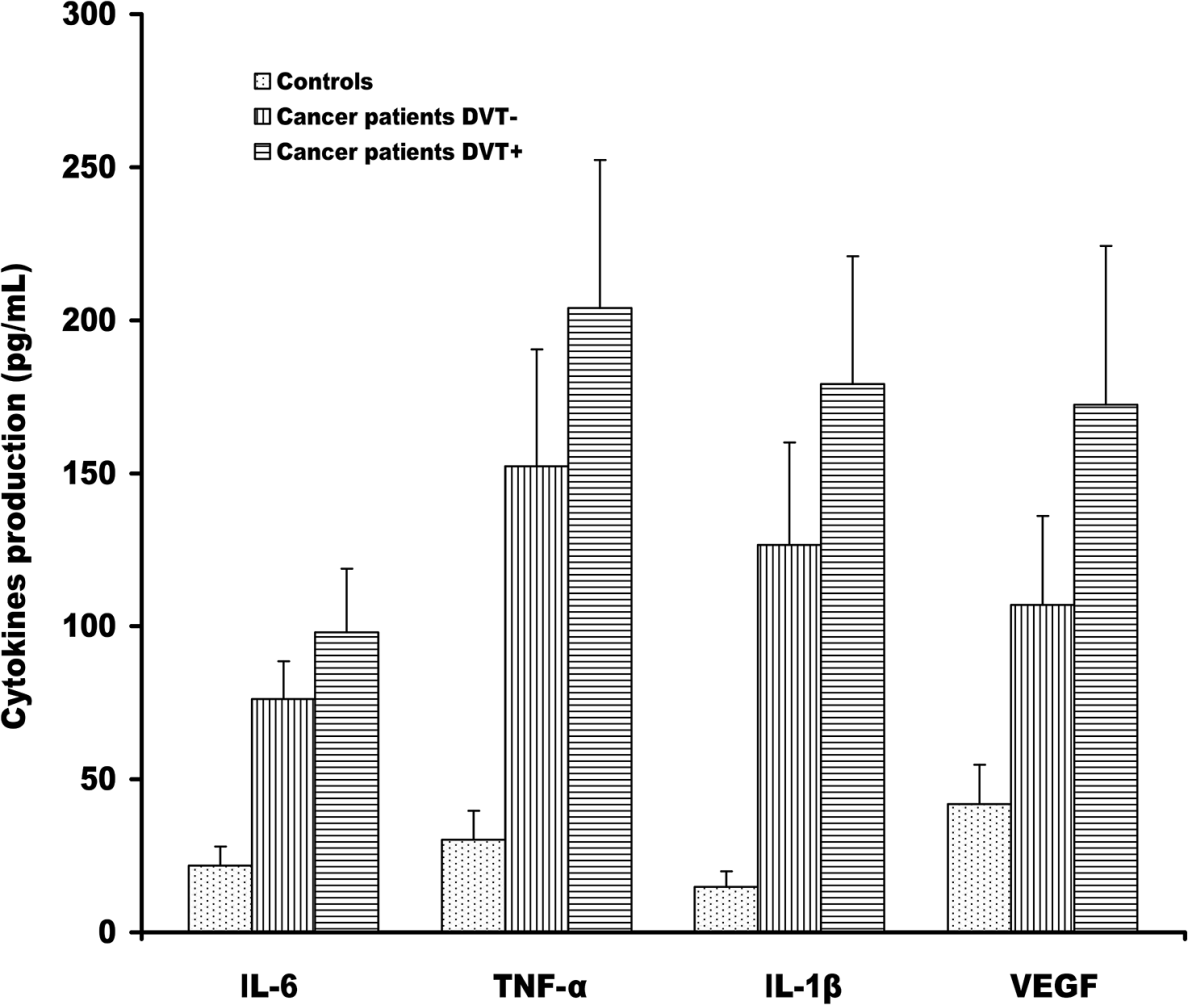
Cytokines secretion in monocytes from cancer patients with and without DVT. Levels of IL-6, TNF-α, IL-1β, and VEGF were measured in supernatants of purified monocytes from cancer patients with and without DVT by a sensitive enzyme-linked immunosorbent assay (ELISA). The results are shown as the means ± SD.

The secretion of these markers analyzed in monocytes supernatant showed similar trend to that observed in plasma. Spontaneous production of cytokines, such as IL-6, TNF-α, Il-1β and VEGF were significantly higher in both groups of cancer patients with and without DVT than those from healthy controls (p<0.0001) (Fig 1). Compared to DVT- cancer patients, DVT+ showed an increased fold change of 1.28 (95% CI: 1.20–1.37; p<0.0001), 1.34 (95% CI: 1.23–1.46; p<0.0001), 1.41 (95% CI: 1.30–1.54; p<0.0001), and 1.61 (95% CI: 1.45–1.78; p<0.0001) for IL-6, TNF-α, Il-1β, and VEGF, respectively. In addition to cytokines, also MMP-9 production and TF activity were higher in DVT+ cancer patients than in those DVT- and in healthy controls (mean ± SD, 103 ± 23, 73 ± 24, 8.8 ± 4, for MMP-9 and mean ± SD, 35.6 ± 6.4, 28 ± 5, 3.3 ± 0.7 for TF activity, respectively). As expected, strong correlations between levels of IL-6, TNF-α, IL-1β, VEGF, MMP-9 and TF in plasma from cancer patients DVT+ and DVT- and those released from monocytes of the same patients were observed (Table 3).

**Table 3:** Spearman correlation coefficients between *in vivo* and *in vitro* marker concentrations. DVT, deep vein thrombosis. A positive strong correlation between the plasma cytokines, angiogenic and coagulation markers and the secretion *in vitro* of the same markers in two groups of cancer patients with and without DVT.

	Cancer patients (n = 321)	Cancer patients +DVT (n = 64)
**IL-6 *vs* IL-6 monocytes**	0.746	0.758
**TNF-α *vs* TNF-α monocytes**	0.684	0.708
**IL-1β *vs* IL-1β monocytes**	0.750	0.772
**MMP-9 *vs* MMP-9 monocytes**	0.728	0.697
**VEGF *vs* VEGF monocytes**	0.805	0.740
**TF antigen *vs* TF activity monocytes**	0.52	0.61

### Effect of DHMEQ on NF-kB p65 subunit activity in monocytes

**Fig 2:**
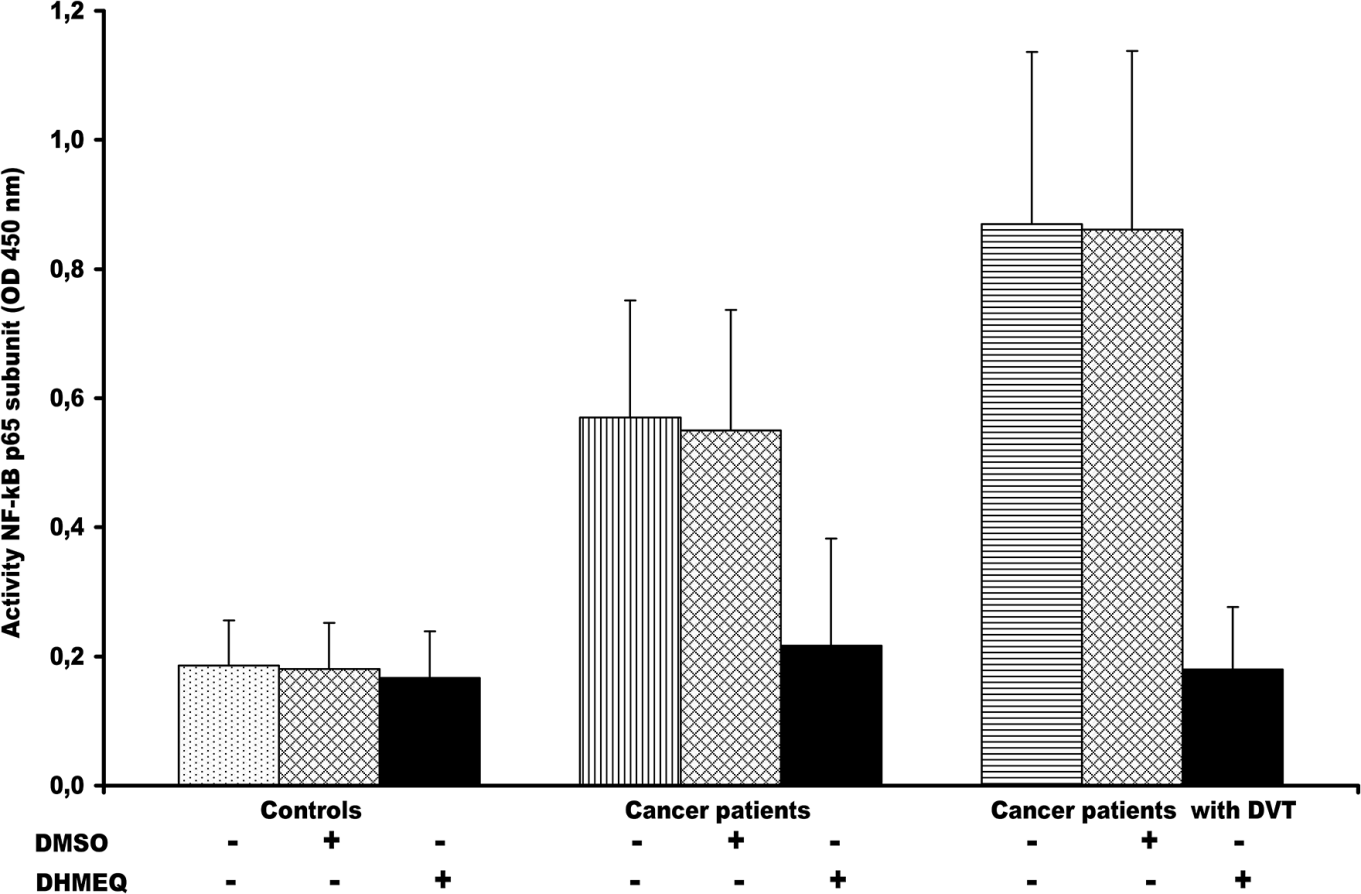
Nuclear factor (NF)–kB p65 subunit activity in monocytes from cancer patients with and without DVT and effect of DHMEQ. The activated NF-kB p65 subunit was significantly higher in cancer patients DVT+ and DVT- than in healthy controls (P< 0.0001). (ANOVA test). NF-kB p65 subunit was significantly higher in cancer patients DVT+ than in those DVT- (P< 0.001) (t-test). To examine the effects of DHMEQ, monocytes were treated with 10 μg/mL DHMEQ. As control experiments, DMSO was used instead of DHMEQ. Monocytes from cancer patients DVT+ were more responsive to DHMEQ than those from DVT-. Intriguingly, no effect of DHMEQ there was in healthy monocytes. The results are shown as the means ± SD. OD, optical density; DVT, Deep Vein thrombosis; DMSO, dimethyl sulfoxide; DHEMQ, dehydroxymethylepoxyquinomicin.

Activation of NF-kB p65 subunit in unstimulated monocytes, from cancer patients DVT+ and DVT- and from healthy control, was analyzed by a sensitive ELISA assay. This assay has the advantage of being 10-fold more sensitive than electrophoresis mobility shift assay (EMSA) and allows greater flexibility in the experimental step. As shown in Fig 2, NF-kB p65 subunit activity in monocytes varied in all examined groups. Higher NF-kB p65 subunit activity was observed in cancer patients DVT+ and DVT- than in healthy controls (p< 0.0001). Significant differences of NF-kB p65 activity were also detected between cancer patients DVT+ and DVT- (p< 0.0001). It was already shown that NF-kB regulates several molecules including those analyzed in the present study [41,42]. Accordingly, all these markers positively correlated with NF-kB activity (Table 4). The identification of these markers may recognize NF-kB as an attractive target for therapeutic intervention. Therefore, we thought to inhibit NF-kB by DHMEQ, a known NF-kB inhibitor [46,47]. The effect of DHMEQ, at the dose of 10 μg/ml, on NF-kB p65 subunit activity inhibition in monocytes from the two groups of cancer patients, was explored (Fig 2). Control experiments with DMSO have been included (Fig 2). Notably, DHMEQ was not effective in monocytes from healthy controls at different dose and time (S1 Fig). Although, DHMQ caused the reduction of cell viability of monocytes derived from cancer patients DVT+ and DVT-, this effect was not observed in monocytes from healthy controls (S2 Fig).

**Table 4:** Correlation between NF-kB p65 activity and inflammation, angiogenetic and thrombotic molecules in monocytes of cancer patients with and without deep vein thrombosis. Abbreviations: DVT, deep vein thrombosis. A positive correlation was found between NF-kB p65 subunit activity and all molecules in cancer patients with and without DVT. Spearman Rank correlation analysis was used.

	Cancer patients (n = 64)	Cancer patients+ DVT (n = 64)
NF-kB vs IL-6	0.66	0.73
NF-kB vs TNF-α	0.58	0.63
NF-kB vs IL-1 beta	0.54	0.57
NF-kB vs MMP-9	0.43	0.64
NF-kB vs VEGF	0.51	0.65
NF-kB vs TF	0.59	0.67

### Effect of LPS and DHMEQ on nuclear factor-kB p65 in monocytes

**Fig 3:**
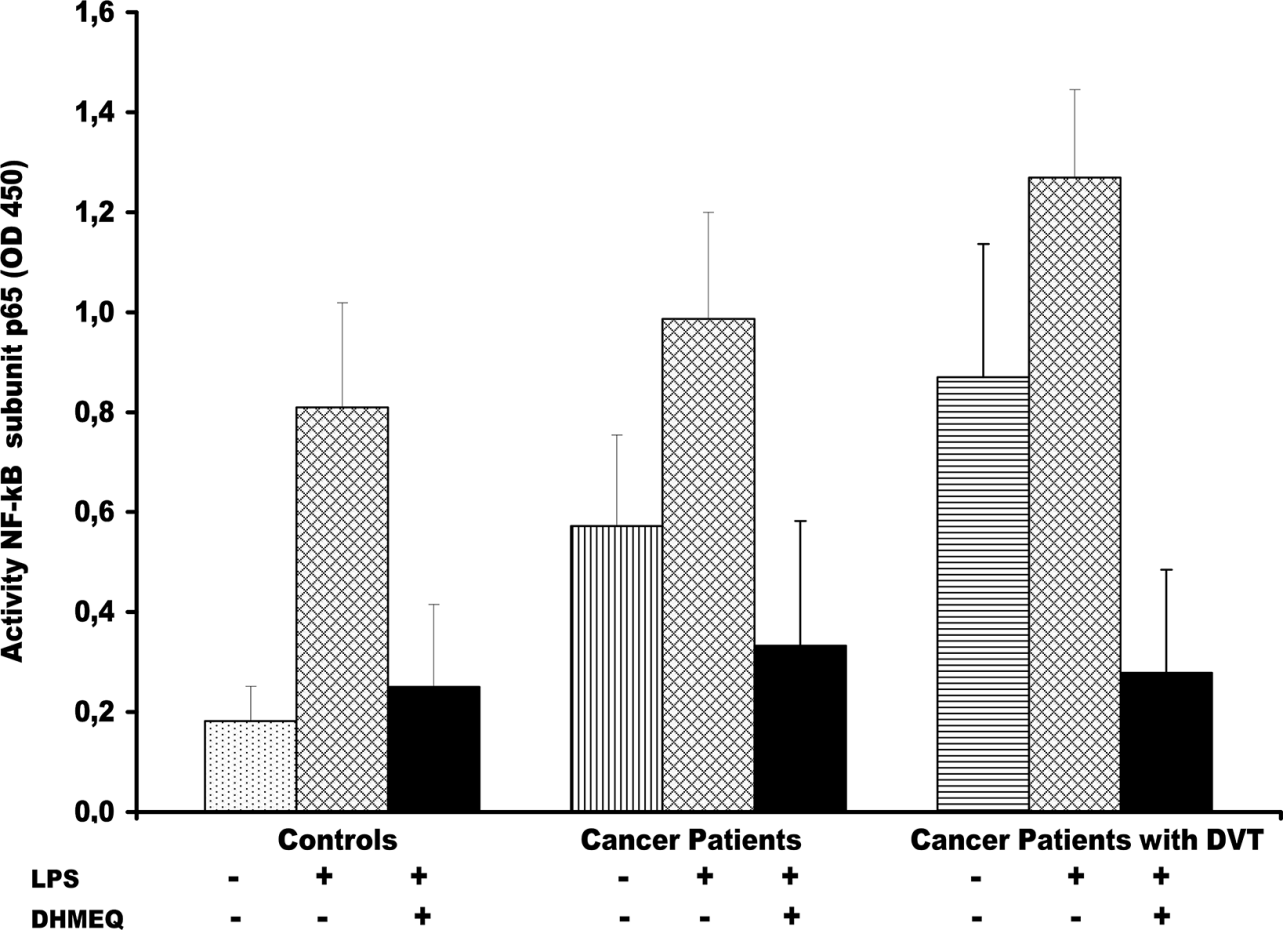
Effect of dehydroxymethylepoxyquinomicin (DHMEQ) on lipopolysaccharide (LPS)-induced nuclear factor (NF)–kB p65 activation in monocytes from cancer patients with and without DVT. Nuclear extracts were prepared from monocytes, pretreated and not with DHMEQ (10 μg/mL) for 2 h and after which stimulated with 100 ng/ml LPS for 24 hour, using a Nuclear Extract Kit. The stimulation of monocytes with LPS at 100 ng/mL induce un significant increase of the nuclear NF-kB p65 protein level in all groups or cancer patients DVT+, DVT- and in healthy controls, compared to unstimulated cells, (P<0.0001). NF-kB p65 subunit was significantly higher in cancer patients DVT+ than in those DVT- (P< 0.0001) (t-test). DVT, Deep Vein thrombosis.

As shown in Fig 3, treatment of monocytes with LPS at 100 ng/mL strongly activated NF-kB p65 of 46%, 73% and 35% over the basal value compared to unstimulated cells in cancer patients DVT+, DVT- and in healthy controls, respectively (p<0.0001). Preincubation with DHMEQ, prior to LPS stimulation, strongly decreased NF-kB p65 subunit activity of 4.5-fold, 3-fold and 3.2-fold in cancer DVT+ and DVT- and in healthy controls (p<0,0001), respectively, compared with LPS alone. To reinforce our findings on NF-kBp65 down-regulation by DHMEQ in LPS-activated monocytes, the amount of NF-kB p65 subunit activity in nuclear extracts from the same experiments were subsequently measured by ELISA. As expected, treatment with DHMEQ in LPS stimulated monocyte induced the decrease of NF-kB activity among the three groups analyzed (Fig 3).

### Effects of DHMEQ on markers release by monocytes

**Fig 4:**
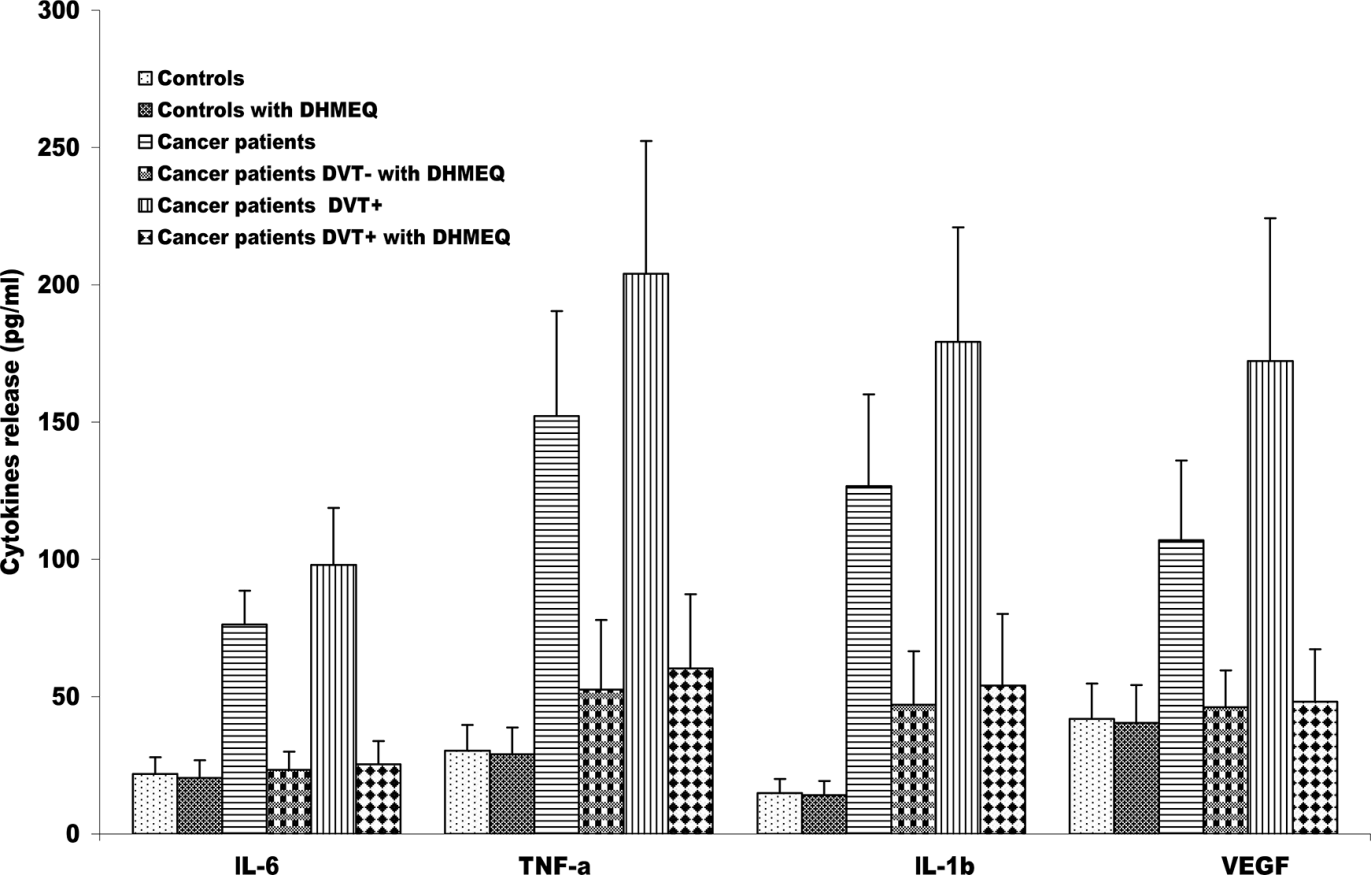
Effects of dehydroxymethylepoxyquinomicin (DHMEQ) on cytokines release by monocytes from cancer patients with and without DVT. Monocytes were treated or not with 10 μg/mL DHMEQ, after which the amounts of interleukins (IL)-6, tumor necrosis factor alpha (TNF-α), IL-1β and vascular endothelial growth factor (VEGF) secreted were measured by enzyme-linked immunosorbent assay (ELISA). Monocytes were incubated for 24 hr. The treatment with DHMEQ induces the decrease of all molecules in both groups of cancer patients with and without DVT compared to untreated cells (P<0.0001), (t-test). The results are shown as the means ± SD. DVT, Deep Vein thrombosis;

DHMEQ treatment was used to asses if NF-kB is directly associated with the release of all markers, detected above, from monocytes of cancer patients DVT+ and DVT-. The Fig 4 shows that treatment with DHMQ dramatically decreases the levels of IL-6, TNF-alpha, IL-1beta and VEGF in both groups of cancer patients compared to untreated cells. Similar trend was observed for MMP-9 and TF. In contrast, no differences were observed in the control group in which constitutive NF-kB activation was absent.

### Serum-dependent activation of NF-kB in healthy monocytes

**Fig 5:**
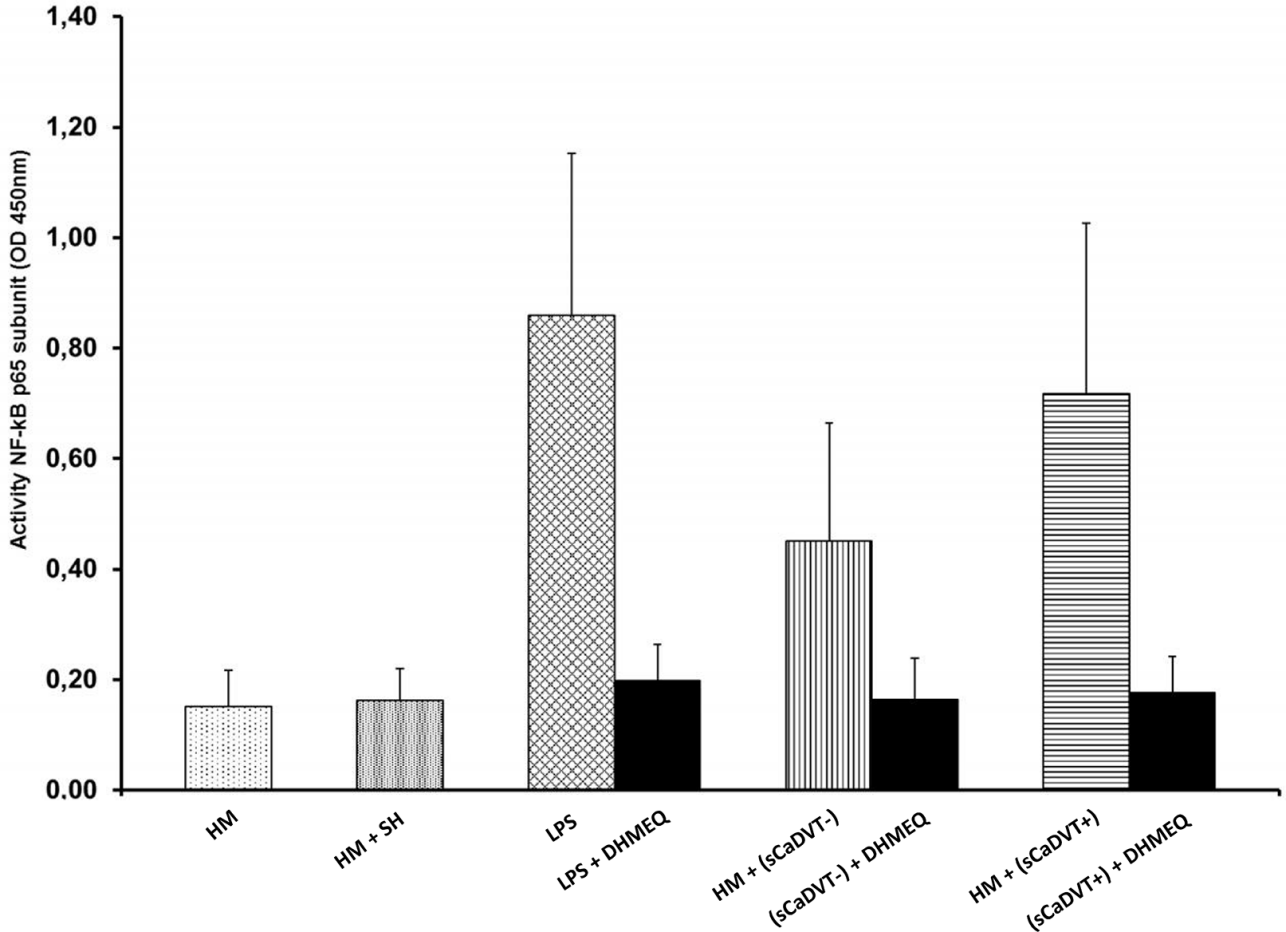
Serum-dependent activation of nuclear factor (NF)–NF-kB p65 subunit in healthy monocytes. Monocytes from 25 healthy controls were evaluated for NF-kB activation after they were cultured for 20 hours in medium supplemented with either 40% serum from three groups. Monocytes (5x10^5^) from 25 healthy donors were cultured for 20 hours in medium (RPMI 1640) supplemented with either 40% serum derived from 64 cancer patients DVT+ and 257 DVT- with the highest cytokines plasma levels (> 75th percentile) or 40% serum derived from 100 healthy donors with the lowest cytokines values (< 25th percentile). The incubation of healthy monocytes with pooled sera derived from cancer patients DVT+ (sCADVT+) or DVT- (sCADVT-) induced a significant increase of NF-kB activity compared with that derived from healthy controls (HM) after treatment with sera from healthy controls (SH) (P<0.0001). An higher NF-kB p65 subunit activation was observed in monocytes stimulated by sera from cancer patients DVT+ compared to that stimulated by sera derived from DVT- (P<0.001) (t-test). No NF-kB p65 subunit activation was observed in monocytes stimulated with sera derived from healthy controls.

As shown in Fig 5, the incubation of healthy monocytes with pooled sera, derived from the cancer patients DVT+ or DVT- with the highest values of cytokines, induced a significant increase of NF-kB activation compared with pooled sera, derived from the healthy controls with the lowest values of the same cytokines, (p<0.0001). In addition, a higher NF-kB activation was observed in monocytes stimulated by sera derived from cancer patients DVT+ compared to that stimulated by sera derived from DVT- (p<0.001) (Fig 5). In order to further exclude the possibility that some LPS contamination might contribute to the NF-kB activation, 10 μg/ml of polymixin B was added in all cell cultures to neutralize any potential LPS contamination. Instead, as positive control of NF-kB activation, LPS was considered. LPS-stimulated cultures induced an higher NF-kB activity statistically significant compared to cancer derived sera-stimulated cultures (p<0.001), while no significant difference was evident between LPS-stimulated cultures and DVT cancer derived sera- stimulated cultures. In the LPS experiments no polymixin B was added. The NF-kB activation, stimulated with the sera derived from cancer patients with and without DVT or LPS, was partially blocked by DHMEQ (10 μg/mL) when added in both cultures and LPS-stimulated cultures.

### Serum-dependent activation of cytokines production in healthy monocytes

In addition to NF-kB activity, we explored the activation of cytokines in healthy monocytes treated with pooled sera obtained from cancer patients DVT+ and DVT- and from healthy controls. Higher mean levels of IL-6, TNF-α, IL-1β, VEGF, MMP-9 and TF were secreted from healthy monocytes treated with the sera derived from cancer patients DVT compared to those from DVT- (p<0.01). On the contrary, healthy monocyte treated with the sera from healthy controls, showed no significant effects (Table 5). When healthy monocytes were incubated with the sera derived from patients with and without DVT in the presence of DHMEQ, the cytokines production strongly decreased compared to untreated cells (p<0.0001) (Table 6).

**Table 5:** Serum-depend activation of NF-kB regulated markers production from healthy monocytes. HM, Monocytes from healthy subjects; sH, sera from healthy subjects; LPS, lipopolysaccharide; sCaDVT-, serum from cancer patients without deep venous thrombosis; sCADVT+, serum from cancer patients with deep venous thrombosis; IL-6, Interleukin-6; TNF- α, Tumor necrosis factor alpha; IL-1β, Interleukin-1 beta; VEGF, Vascular endothelial growth factor; MMP-9, matrix metalloproteinase-9; TF, Tissue factor. All p values were calculated by Wilcoxon Matched-Pairs Signed-Ranks Test.

	HM	HM + sH	P value°	HM + sCa_DVT-_	HM + sCa_DVT+_	P value*
**IL-6**	21.1 ± 6.1	21 ± 7.1	ns	66.4 ± 25.4	82.3 ± 25.9	0.01
**TNF-α**	30.3 ± 9.4	29.2 ± 9.2	ns	121 ± 44.2	143 ± 43.6	0.01
**IL-1β**	15 ± 5.1	14 ± 5.9	ns	80.2 ± 27.2	111 ± 34.9	0.01
**VEGF**	42 ± 12.8	36 ± 11.6	ns	98.8 ± 17.6	121 ± 28.7	0.01
**MMP-9**	8.8 ± 3.7	9.4 ± 4.9	ns	58.4 ± 12.4	70.9 ± 17	0.01
**TF**	2.7 ± 0.8	2.5 ± 0.5	ns	57.2 ± 14.7	85.4 ± 18.6	0.01

(°) p value calculated between HM and (HM+sH)

(*) p value calculated between (HM + sCaDVT-) and (HM + sCaDVT+).

**Table 6:** Serum-depend activation of NF-kB regulated markers production from healthy monocytes treated with DHMEQ. HM, Monocytes from healthy subjects; sH, sera from healthy subjects; LPS, lipopolysaccharide; sCaDVT-, serum from cancer patients without deep venous thrombosis; sCADVT+, serum from cancer patients with deep venous thrombosis; IL-6, Interleukin-6; TNF- α, Tumor necrosis factor alpha; IL-1β, Interleukin-1 beta; VEGF, Vascular endothelial growth factor; MMP-9, matrix metalloproteinase-9; TF, Tissue factor. All p values were calculated by Wilcoxon Matched-Pairs Signed-Ranks Test.

	HM + sCa_DVT-_	HM + sCa _DVT-_+ DHMEQ	P value^$^	HM + sCa_DVT+_	HM + sCa_DVT+_ DHMEQ	P value^£^
IL-6	66.4 ± 25.4	19.4 ± 9	0.0001	82.3 ± 25.9	22 ± 7.1	0.0001
TNF-α	121 ± 44.2	30.7 ± 9.2	0.0001	143 ± 43.6	32 ± 9.7	0.0001
IL-1β	80.2 ± 27.2	15.2 ± 6.4	0.0001	111 ± 34.9	16.2 ± 7.3	0.0001
VEGF	98.8 ± 17.6	35 ± 10	0.0001	121 ± 28.7	37 ± 11	0.0001
MMP-9	58.4 ± 12.4	10.2 ± 5.5	0.0001	70.9 ± 17	11 ± 5.1	0.0001
TF	57.2 ± 14.7	2.8 ± 0.8	0.0001	85.4 ± 18.6	3.1 ± 1.2	0.0001

(^$^) p value calculated between (HM + sCaDVT-) and (HM + sCa DVT-+ DHMEQ)

(^£^) (HM + sCaDVT+) and (HM + sCaDVT+ DHMEQ)

Control experiments, conducted with LPS, indicated that a remarkable significant enhancement of all markers production, compared to those stimulated with the sera derived from cancer patients DVT- (p<0.0001). As expected, no significant differences were observed in the production of all markers between the group of healthy monocytes treated with LPS and that treated with the sera derived from cancer patients DVT+ (S2 Table).

## Discussion

Patients with cancer can experience complications including thrombosis, bleeding, and disseminated intravascular coagulation. The identification of novel therapeutic targets in cancer patients with high risk of DVT may encourage further protective studies to improve the management of these patients. To our knowledge, this is the first report that analyzes multiple NF-kB-dependent markers in cancer patients with and without DVT.

In addition, protein secretion was also tested in order to find out whether the changes in the cytokine plasma levels were associated with changes in cytokines production. As expected, the plasma levels of markers analyzed were upregulated in the cancer patients with and without DVT compared to healthy controls, although they were statistically higher in DVT+, with the exception of fibrinogen that was similar in both groups. The cytokines measured in our experiments are largely involved in the upregulation of inflammatory reactions and thus in numerous malignancies. When we have investigated the protein's secretion, the same plasma trend was also observed in monocytes of patients. We have found an increasing release of inflammatory cytokines, MMP-9 and VEGF in monocytes from two groups of cancer patients with the highest release levels in the group with DVT. As expected, the involvement of tumor microenvironment in cancer and in its complications, such as DVT, is emphasized in the correlation between plasma levels of IL-6,TNF-α, IL-1β, VEGF and MMP-9 from cancer patients with and without DVT and those released from monocytes. In addition, mTF activity was very high in cancer patients with DVT. It is an important coagulation factor that has been reported in many types of cancers [48,49]. Furthermore, mTF activity in our patients linearly correlated with TF antigen and other inflammatory and angiogenic markers *in vivo and in vitro*. Cytokines such as, IL-6, TNF-α and IL-1β have been shown to be able to increase tumor cell pro-coagulant activity [50–52], enhancing clotting activation in cancer patients.

Therefore, increased production of all these markers, found in the present study, may result as activation of host cells, such as monocytes, present in tumor environment, and/or of cytokines released by tumor cells themselves. Malignant cells can directly activate blood coagulation by regulating fibrinogen, tissue factor, cancer procoagulant activity, inflammatory reactions and cytokines [53]. These findings are in agreement with previous studies, demonstrating increased plasmatic or cellular release in patients with different tumor types [21,23,54–60], and in those with DVT [28,61–64].

As previously mentioned, monocytes of two groups of cancer patients produced high levels of CRP, TNF-α and IL-1β, that may stimulate NF-kB activity by an autocrine mechanism [42]. A growing body of studies suggests that NF-kB activation mediates up-regulation of inflammatory cytokines, angiogenic factors (e.g. CRP, IL-6, TNF-α, IL-1β, VEGF, MMP-9) in cancer [41,65–69]. Administration of selective inhibitors of the NF-kB pathway can sensitize tumor cells to reduce the release of cytokines [47,70,71]**.** We have, first, observed that the activity on NFkB p65 subunit in our patient groups examined was higher when compared with controls. Next, we evaluated the effect of the DHMEQ, a specific inhibitor of NF-kB, to determine whether NF-kB inhibition was associated with the decrease of IL-6,TNF-a, IL-1β, VEGF, MMP-9 and mTF activity. As expected, all inflammatory, angiogenic and thrombotic cytokine, were significantly reduced when the monocytes of patients were treated with DHMEQ. These observation are in agreement with recent in vitro studies [72,73].

Tumor microenvironment, in our patient groups, may be responsible of NFkB activation [41,67,74]. To further investigate the inhibitory mechanism of DHMEQ action, we examined its effect on monocytes LPS-mediated NF-kB activation. A precise monitoring of NF-kB activation in cells of cancer patients with and without DVT is essential for signal transduction pathway analysis. In this study, we also showed that a constitutive activation of NF-kB p65 subunit was more elevated in cancer patients with DVT than in cancer patients without DVT; NF-kB activation was not evident in healthy controls. In addition, we have observed that LPS-induced NF-kB activation was significantly reduced in DHMEQ pretreated monocytes from cancer patients with and without DVT and in healthy controls.

A correlation among NF-kB and inflammatory, angiogenic and thrombotic markers, here observed, suggest that NF-kB activation is considered an amplifying and perpetuating mechanism of the inflammation, angiogenic and thrombotic process. Accordingly, it was shown that NF-kB regulates the expression of many genes producing, cytokines, chemokines and adhesion molecules involved in the cascade of both inflammation and coagulation [51,75–77]. Tumor cells themselves can induce the expression of TF by host cells such as endothelial cells or monocytes/macrophages [78]. Thus, TF on tumor or host cell surface represent a major mediator of clotting activation at the tumor-host interface.

To further demonstrated if NF-kB activation was mediated by the several molecules involved in both cancer and DVT development, we used a pool of sera from cancer patients with and without DVT to treat the monocyte derived from healthy donors. The results suggest the significant role played by all molecules, detected in cancer patients, that are able to activate NF-kB pathway that in turn enhance the production of inflammatory, angiogenic and thrombotic markers.

Overall, the results of the present study suggest that the inhibition of NF-kB by DHMEQ reduces the positive inflammatory, angiogenic and thrombotic loop generated by multiple markers detected in both cancer and DVT. Therefore, our data support the notion that NF-kB transcription factor may be considered as an excellent therapeutic target for cancer patients, especially those complicated by DVT and treatment with NF-kB inhibitors may corroborate the efficacy of chemotherapeutic agents.

## Supporting Information

S1 Fig Inhibition of NF-kB p65 subunit activity by DHMEQ in monocytes. **Dose and time dependent manner.** (A) Dose-dependent reduction of NF-kB p65 subunit activity was observed in monocytes treated with DHMEQ. Monocytes from cancer patients DVT+ and DVT- and those from healthy volunteers were treated at the indicated concentrations of DHMEQ and time points. (B) Time course analyses of NF-kB p65 subunit activity in monocytes from three groups analyzed treated with DHMEQ. NF-kB p65 subunit activity was measured by ELISA. Data represent the mean ± SD in the 3 groups of individuals.
(TIF)

S2 Fig Cell viability of monocytes after treatment with dehydroxymethylepoxyquinomicin (DHMEQ). (A) Dose-dependent reduction of cell viability of monocytes treated with DHMEQ. Monocytes from cancer patients with and without DVT and those of healthy volunteers, used as controls, were treated at the indicated concentrations of DHMEQ and time points. Cell viability were determined by MTT assay. (B) Time course analyses of cell viabilities of monocytes from three groups analyzed treated with DHMEQ. Data represent the mean ± SD of relative viabilities of 3 independent experiments.
(TIF)

S1 Table Spearman correlation coefficients among plasma concentrations. Note: CRP, C Reactive Protein, IL-1β, Interleukin-1 beta; IL-6, Interleukin-6; TNF- α, Tumor necrosis factor-alpha; MMP-9, matrix metalloproteinase-9; VEGF, Vascular endothelial growth factor; TF, Tissue factor; sP, Spearman correlation.
(DOC)

S2 Table Serum-depend activation of NF-kB regulated markers production from healthy monocytes treated with LPS. Note: HM, Monocytes from healthy subjects; sH, sera from healthy subjects; LPS, lipopolysaccharide; sCaDVT-, serum from cancer patients without deep venous thrombosis; sCADVT+, serum from cancer patients with deep venous thrombosis; IL-6, Interleukin-6; TNF- α, Tumor necrosis factor alpha; IL-1β, Interleukin-1 beta; VEGF, Vascular endothelial growth factor; MMP-9, matrix metalloproteinase-9; TF, Tissue factor. All p values were calculated by Wilcoxon Matched-Pairs Signed-Ranks Test. (§) p value calculated between (HM + LPS) and (HM + sCaDVT-); (^) p value was calculated between (HM + LPS) and (HM + sCaDVT+).
(DOCX)
