# Supplementary figures and images for: Increased Levels of NF-kB-Dependent Markers in Cancer-Associated Deep Venous Thrombosis
Source: PLoS One. 2015 Jul 20;10(7):e0132496. doi: 10.1371/journal.pone.0132496 (PMC4507873; doi:10.1371/journal.pone.0132496)

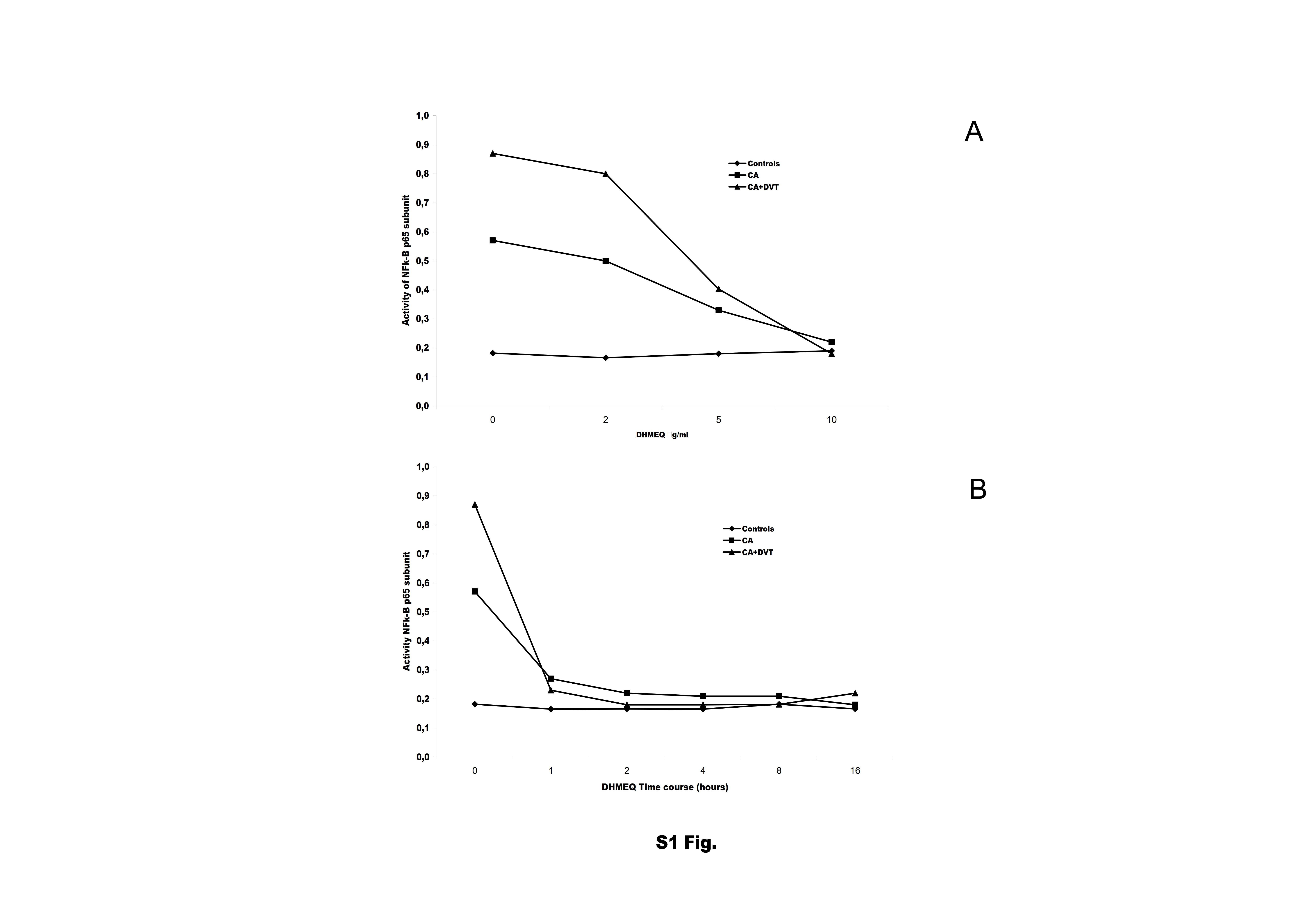

Supplement: S1 Fig — Dose and time dependent manner. (A) Dose-dependent reduction of NF-kB p65 subunit activity was observed in monocytes treated with DHMEQ. Monocytes from cancer patients DVT+ and DVT- and those from healthy volunteers were treated at the indicated concentrations of DHMEQ and time points. (B) Time course analyses of NF-kB p65 subunit activity in monocytes from three groups analyzed treated with DHMEQ. NF-kB p65 subunit activity was measured by ELISA. Data represent the mean ± SD in the 3 groups of individuals. (TIF) [file pone.0132496.s001.tif]

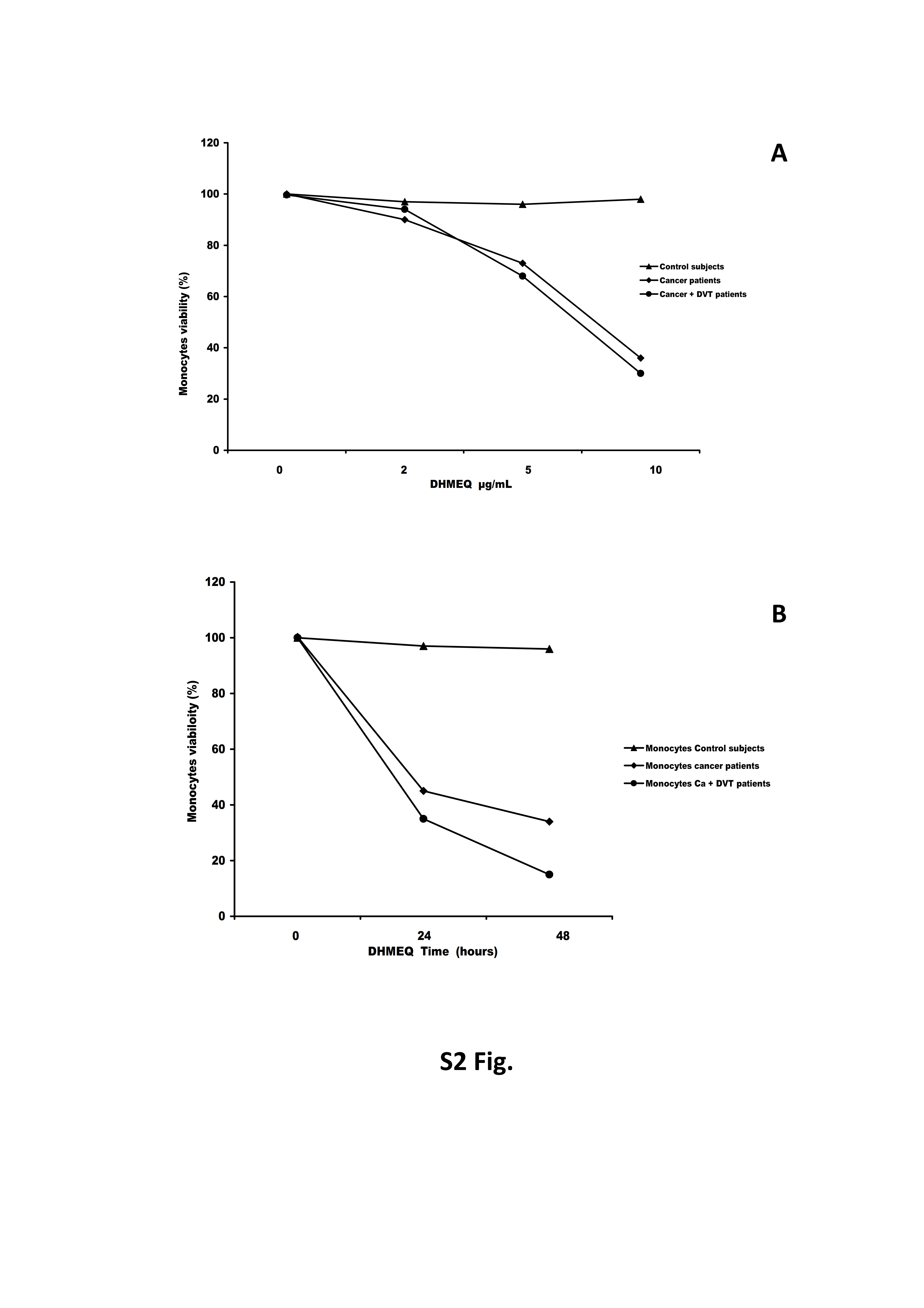

Supplement: S2 Fig — (A) Dose-dependent reduction of cell viability of monocytes treated with DHMEQ. Monocytes from cancer patients with and without DVT and those of healthy volunteers, used as controls, were treated at the indicated concentrations of DHMEQ and time points. Cell viability were determined by MTT assay. (B) Time course analyses of cell viabilities of monocytes from three groups analyzed treated with DHMEQ. Data represent the mean ± SD of relative viabilities of 3 independent experiments. (TIF) [file pone.0132496.s002.tif]
