# Supplementary material for: Increased Levels of NF-kB-Dependent Markers in Cancer-Associated Deep Venous Thrombosis
Source: PLoS One. 2015 Jul 20;10(7):e0132496. doi: 10.1371/journal.pone.0132496 (PMC4507873; doi:10.1371/journal.pone.0132496)
Supplement: S1 Table — Note: CRP, C Reactive Protein, IL-1β, Interleukin-1 beta; IL-6, Interleukin-6; TNF- α, Tumor necrosis factor-alpha; MMP-9, matrix metalloproteinase-9; VEGF, Vascular endothelial growth factor; TF, Tissue factor; sP, Spearman correlation. (DOC) [file pone.0132496.s003.doc]

**Supplementary Table 1. Spearman correlation coefficients among plasma concentrations**

Cancer cases with thrombosis (n=64)

|  | **Fibrinogen** | **CRP** | **IL-1β** | **IL-6** | **TNF-α** | **MMP-9** | **VEGF** | **FT** | **sP** |
| --- | --- | --- | --- | --- | --- | --- | --- | --- | --- |
| **Fibrinogen** | 1 | 0.584 | 0.546 | 0.602 | 0.510 | 0.515 | 0.471 | 0.512 | 0.399 |
| **CRP** |  | 1 | 0.678 | 0.745 | 0.600 | 0.695 | 0.657 | 0.711 | 0.458 |
| **IL-1β** |  |  | 1 | 0.696 | 0.765 | 0.742 | 0.609 | 0.783 | 0.641 |
| **IL-6** |  |  |  | 1 | 0.681 | 0.747 | 0.789 | 0.671 | 0.628 |
| **TNF-α** |  |  |  |  | 1 | 0.652 | 0.585 | 0.655 | 0.610 |
| **MMP-9** |  |  |  |  |  | 1 | 0.630 | 0.611 | 0.574 |
| **VEGF** |  |  |  |  |  |  | 1 | 0.658 | 0.670 |
| **FT** |  |  |  |  |  |  |  | 1 | 0.666 |
| **sP** |  |  |  |  |  |  |  |  | 1 |

**Cancer ca**ses without thrombosis (n=321)

|  | **Fibrinogen** | **CRP** | **IL-1β** | **IL-6** | **TNF-α** | **MMP-9** | **VEGF** | **FT** | **sP** |
| --- | --- | --- | --- | --- | --- | --- | --- | --- | --- |
| **Fibrinogen** | 1 | 0.096 | 0.099 | 0.058 | 0.150 | 0.066 | 0.067 | 0.091 | 0.139 |
| **CRP** |  | 1 | 0.656 | 0.710 | 0.614 | 0.527 | 0.511 | 0.586 | 0.454 |
| **IL-1β** |  |  | 1 | 0.660 | 0.691 | 0.537 | 0.544 | 0.670 | 0.578 |
| **IL-6** |  |  |  | 1 | 0.592 | 0.707 | 0.621 | 0.644 | 0.559 |
| **TNF-α** |  |  |  |  | 1 | 0.562 | 0.517 | 0.606 | 0.537 |
| **MMP-9** |  |  |  |  |  | 1 | 0.623 | 0.596 | 0.531 |
| **VEGF** |  |  |  |  |  |  | 1 | 0.573 | 0.602 |
| **FT** |  |  |  |  |  |  |  | 1 | 0.572 |
| **sP** |  |  |  |  |  |  |  |  | 1 |

Healthy controls (n=100)

|  | **Fibrinogen** | **CRP** | **IL-1β** | **IL-6** | **TNF-α** | **MMP-9** | **VEGF** | **FT** | **sP** |
| --- | --- | --- | --- | --- | --- | --- | --- | --- | --- |
| **Fibrinogen** | 1 | 0.045 | -0.019 | 0.009 | 0.094 | 0.035 | -0.119 | -0.097 | -0.088 |
| **CRP** |  | 1 | -0.005 | 0.166 | 0.089 | -0.093 | 0.140 | 0.066 | -0.037 |
| **IL-1β** |  |  | 1 | -0.172 | -0.094 | 0.007 | -0.167 | -0.026 | 0.107 |
| **IL-6** |  |  |  | 1 | 0.098 | 0.006 | 0.142 | 0.055 | 0.090 |
| **TNF-α** |  |  |  |  | 1 | -0.010 | -0.129 | -0.168 | 0.177 |
| **MMP-9** |  |  |  |  |  | 1 | -0.001 | -0.181 | -0.113 |
| **VEGF** |  |  |  |  |  |  | 1 | 0.203 | -0.159 |
| **FT** |  |  |  |  |  |  |  | 1 | -0.028 |
| **sP** |  |  |  |  |  |  |  |  | 1 |
