# Supplementary material for: Increased Levels of NF-kB-Dependent Markers in Cancer-Associated Deep Venous Thrombosis
Source: PLoS One. 2015 Jul 20;10(7):e0132496. doi: 10.1371/journal.pone.0132496 (PMC4507873; doi:10.1371/journal.pone.0132496)
Supplement: S2 Table — Note: HM, Monocytes from healthy subjects; sH, sera from healthy subjects; LPS, lipopolysaccharide; sCaDVT-, serum from cancer patients without deep venous thrombosis; sCADVT+, serum from cancer patients with deep venous thrombosis; IL-6, Interleukin-6; TNF- α, Tumor necrosis factor alpha; IL-1β, Interleukin-1 beta; VEGF, Vascular endothelial growth factor; MMP-9, matrix metalloproteinase-9; TF, Tissue factor. All p values were calculated by Wilcoxon Matched-Pairs Signed-Ranks Test. (§) p value calculated between (HM + LPS) and (HM + sCaDVT-); (^) p value was calculated between (HM + LPS) and (HM + sCaDVT+). (DOCX) [file pone.0132496.s004.docx]

**Supplementary Table 2.** Serum-depend activation of NF-kB regulated markers production from healthy monocytes treated with LPS

|  | **HM+ LPS** | **HM + sCa_DVT-_** | **P value^§^** | **HM + sCa_DVT+_** | **P value^** |
| --- | --- | --- | --- | --- | --- |
| **IL-6** | 141 ± 81.1 | 66.4 ± 25.4 | 0.01 | 82.3 ± 25.9 | ns |
| **TNF-α** | 214 ± 114.5 | 121 ± 44.2 | 0.01 | 143 ± 43.6 | ns |
| **IL-1β** | 143 ± 46.6 | 80.2 ± 27.2 | 0.01 | 111 ± 34.9 | ns |
| **VEGF** | 150 ± 48.7 | 98.8 ± 17.6 | 0.01 | 121 ± 28.7 | ns |
| **MMP-9** | 92.4 ± 45.4 | 58.4 ± 12.4 | 0.01 | 70.9 ± 17 | ns |
| **TF** | 120 ± 44.8 | 57.2 ± 14.7 | 0.01 | 85.4 ± 18.6 | ns |
